# Bacteriophage Treatment: Critical Evaluation of Its Application on World Health Organization Priority Pathogens

**DOI:** 10.3390/v13010051

**Published:** 2020-12-30

**Authors:** Raghad Khalid AL-Ishaq, Sini Skariah, Dietrich Büsselberg

**Affiliations:** Weill Cornell Medicine-Qatar, Education City, Qatar Foundation, Doha 24144, Qatar; rkmalishaq@hotmail.com (R.K.A.-I.); sis2013@qatar-med.cornell.edu (S.S.)

**Keywords:** phage therapy, *S. aureus*, *K. pneumoniae*, *P. aeruginosa*, *E. faecalis*, combination therapy

## Abstract

Bacteriophages represent an effective, natural, and safe strategy against bacterial infections. Multiple studies have assessed phage therapy’s efficacy and safety as an alternative approach to combat the emergence of multi drug-resistant pathogens. This systematic review critically evaluates and summarizes published articles on phages as a treatment option for *Staphylococcus aureus*, *Klebsiella pneumoniae*, *Pseudomonas aeruginosa*, and *Enterococcus faecalis* infection models. It also illustrates appropriate phage selection criteria, as well as recommendations for successful therapy. Published studies included in this review were identified through EMBASE, PubMed, and Web of Science databases and were published in the years between 2010 to 2020. Among 1082 identified articles, 29 studies were selected using specific inclusion and exclusion criteria and evaluated. Most studies (93.1%) showed high efficacy and safety for the tested phages, and a few studies also examined the effect of phage therapy combined with antibiotics (17.2%) and resistance development (27.6%). Further clinical studies, phage host identification, and regulatory processes are required to evaluate phage therapy’s safety and efficacy and advance their clinical use.

## 1. Introduction

Bacteriophages are non-living biological entities consisting of genetic material (DNA or RNA) enclosed within a protein capsid capable of infecting and replicating within bacterial cells [1]. They were first identified in 1915 by Frederick Twort and considered the most abundant organism on Earth, with estimated numbers ranging from 10^31^–10^32^ [2]. Bacteriophages play significant roles in microbial dynamics, physiology, evolution, and therapeutics [3]. They are naturally occurring bacterial parasites, depending on the bacterial host for survival, and are incapable of reproducing independently. They replicate through two primary life cycles, the lytic cycle, where phages infect and rapidly kill their bacterial host, or the lysogenic cycle, where they either integrate their genome into the infected host cells (prophages) or exist as plasmids within the bacterial host [4].

The emergence of antibiotic resistance organisms poses a fundamental threat to public health worldwide. Because of that, phage therapy presents a promising alternative approach to combat emerging pathogens [5]. Bacteriophage (phage) therapy is defined as administering whole lytic phage or purified phage particles directly to a patient to lyse the bacterial pathogen that is causing the infection [6]. Although the practice of phage therapy has been around for a century, the idea of therapeutically using phages against bacterial infections has recently gained attention in response to the emergence of antibiotic resistance pathogens [3]. Advantages of using phages as treatment include (i) high specificity with most phages infecting only a single species of bacteria, (ii) low natural toxicity as they kill targeted bacteria without disrupting the host’s normal flora or human cells, (iii) are unlikely to induce cross-resistance to antibiotics [7], (iv) anti-biofilm activity with bacteriophages in contrast to most antibiotics being able to penetrate and disperse existing biofilms and in some cases even prevent further biofilm formation [8], and (v) the presence of massive untapped natural repertoire of diverse bacteriophages offering numerous treatment options and potential combination cocktails. Phage therapy is effective against both antibiotic resistance and antibiotic sensitive pathogens. Their activity is not simply bacteriostatic but rather bactericidal, thus eradicating their target pathogens and thereby preventing bacterial evolution towards resistance [9]. Additionally, phage therapy also reverses antibiotic resistance and restore susceptibility to various classes of antibiotic in *Pseudomonas aeruginosa* [10].

In this systematic review, we evaluated and analyzed 29 published articles on phages targeting selected key pathogens that report on their antimicrobial activity and their feasibility of these being used as alternative treatment options. The selected pathogens are *S. aureus, K. pneumoniae, P. aeruginosa*, and *E. faecalis.* They were chosen from the World Health Organization (WHO) priority list. *S. aureus* and *E. faecalis* are Gram-positive bacteria and major human pathogens that cause a wide range of clinical manifestations [11]. They can cause bacteremia, sepsis, skin and soft tissue infections, and urinary tract infections depending on the site of infection and the strains involved [12]. On the other hand, *K. pneumoniae* and *P. aeruginosa* are Gram-negative bacteria with polysaccharide capsules that allow bacteria to evade the immune system and cause fatal bacteremia and pneumoniae [13,14]. Mechanisms of immune evasion in these bacteria include antiphagocytic capsule production, biofilm formation, and intracellular survival [15]. The reasons behind choosing these pathogens were (i) they represent top critical and high category WHO priority pathogens against which there is an urgent need for the development of new therapeutic options, (ii) to test the applicability of phage therapy against both Gram-positive and Gram-negative pathogens and drug-resistant pathogens. For this reason, two Gram-negative and two Gram-positive bacteria with diverse antibiotic resistance profiles were chosen, which might help in better understanding of the broad applicability of the phages, and (iii) to bridge the lack of protocol standardization in clinical applications of phages as we have identified gaps in the current literature.

## 2. Material and Method

### 2.1. Search Methods

**Figure 1:**
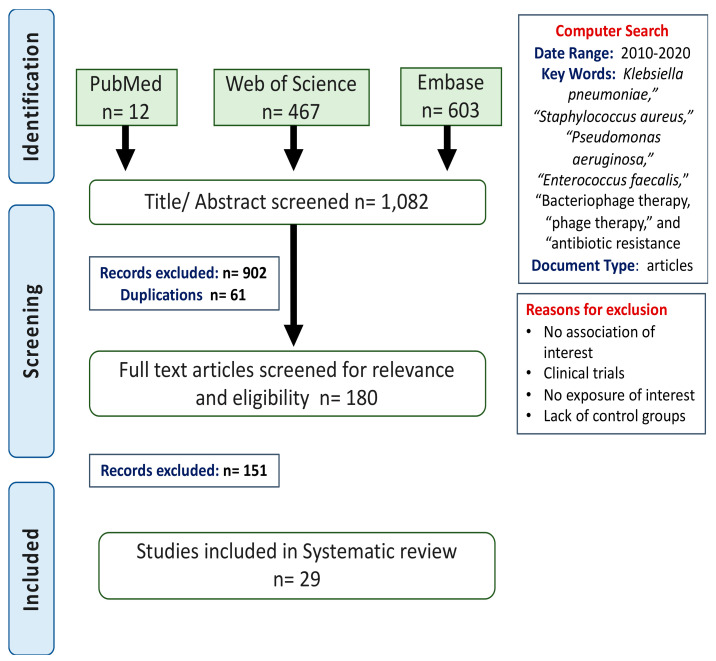
Flow diagram of search process. The diagram is divided into three steps; identification, screening, and articles included. Search terms and date range were identified in the computer search box. Eligible studies included in vivo, and in vitro models infected with one of the four selected pathogens and were treated with bacteriophage specific to the pathogen. All clinical trials were excluded. Abbreviations; n: number of studies.

A comprehensive literature search was done on EMBASE, Web of Science, and PubMed databases for articles on phage therapy that were published from 2010 to 2020. Searched terms included “*Klebsiella pneumoniae*”, “*Staphylococcus aureus*”, “*Pseudomonas aeruginosa*”, “*Enterococcus faecalis*”, “Bacteriophage therapy, “phage therapy”, and “antibiotic resistance”. Figure 1 illustrates flow diagram describing the search process.

### 2.2. Study Selection and Data Collection

Various publications identified in the search were further analyzed more closely to determine eligibility for further inclusion in this systematic review. Duplicate studies were excluded, and eligible studies were selected based on inclusion and exclusion criteria. Inclusion criteria included in vivo and in vitro studies where models infected with one of the four chosen pathogens were treated with bacteriophages specific to the pathogen. Studies with models infected with other pathogens and all clinical trials were excluded. Data retrieved from the 29 eligible studies include: model used, type of infection, target pathogen, phage/cocktail identification, method of phage inoculation, phage doses, treatment duration, phage safety and efficacy, the use of antibiotics to evaluate combination therapy, and if available the emergence of bacterial or phage resistance. Table 1 summarizes the retrieved information from the 29 reviewed articles.

### 2.3. Critical Evaluation

The primary outcomes of interest were the efficacy and safety of using phage therapy against one or more of the selected four pathogens. The selected studies were evaluated to determine whether phages were (i) effective as indicated by the phage ability to reduce bacterial concentration, alleviate or cure the infection in the model used, (ii) safe as illustrated by the absence of inflammation and any side effects related to phage application, and (iii) polyvalent indicated by the ability of phage to target and kill multiple bacterial strains. The secondary outcomes of interest were the co-effect of phage combined with antibiotics as a treatment option and the absence of bacterial resistance to the phage used.

## 3. Results

### 3.1. Study Characteristics

Our initial search yielded 1082 articles (*S. aureus*; 531, *K. pneumonia*; 125, *E. faecalis*; 72, and *P. aeruginosa*; 354 articles). After reviewing and eliminating studies based on inclusion and exclusion criteria, we critically reviewed the full-text manuscripts of 29 studies. The specific details regarding infection and treatment, including the outcome for these studies are listed in Table 1. Most studies (75.9%) investigated the use of single phage rather than cocktails. In 5 studies, phages were used combined with antibiotics as a therapy approach; all others tested phages as a monotherapy. Animal/biofilm models used in the studies received phage doses ranging from 10^4^ to 10^10^ plaque-forming units (PFU) per dose. The duration of phage treatment follows up in models ranged from one hour to thirty days.

### 3.2. Characteristics of Model Used

Out of the selected 29 studies, 18 studies used multiple in vivo models that were used to address and test the hypothesis, such as Albino mice (5.6%), BALB/C mice (50%), Swiss webster mice (5.5%), Sprague-Dawley rats (11.2%), rabbit (5.5%), C57BL/6 mice (11.2%), Wistar rats (5.5%), and 5.5% of the selected articles used unspecified mice. These models’ average age ranged from one month to twenty weeks, with females being the predominant gender (eight studies used male mice). For the remaining eleven studies that assessed biofilm/bacterial growth, researchers used in vitro models in usually 96 well polyester tissue culture plates and test flasks to conduct their experiments. Animal or biofilm models were infected with *S. aureus* (24.1%), *K. pneumonia* (17.2%), *E. faecalis* (20.7%), and *P. aeruginosa* (27.6%). Two studies investigated both *S. aureus* and *P. aeruginosa* and one study observed the effect of phage therapy on *K. pneumonia*, *S. aureus,* and *P. aeruginosa.* Although the administration of phage therapy as a cocktail or single phage was done intraperitoneally in nine studies, five studies evaluated a novel intranasal approach as an alternative administration method for pneumonia treatment, one study each investigated the intravenous approach and oral delivery respectively, and in two studies a subcutaneous injection or through the skin mode was used for phage delivery. One study tested both intraperitoneal and intragastric inoculation. In the eleven exclusive in vitro studies, phages were added directly into the in vitro models.

### 3.3. Phage Isolation and Purification Protocol

Isolation and purification of bacteriophages are critical steps that can determine phage’s utility for therapy. Although the basic and the most common method used for phage isolation is the enrichment procedure, some researchers isolate phage by directly plating environmental samples and look for plaque-forming units or spot culturing. For the enrichment procedure, the environmental source samples are rid of endogenous bacteria by centrifugation or filtration and then incubated with the target bacterial sample to assess for the presence of phage(s) [45].

In the 29 studies reviewed, bacteriophages were isolated majorly from sewage (55.2.%), and they were mainly detected and purified using a double-layer agar plate (DLA) and picking single plaques and re-plating multiple times. Phage concentration and purification majorly started with an enrichment step/culture step involving incubation with host bacteria, followed by centrifugation to eliminate debris and bacteria and or filtration or chloroform treatment to remove residual bacteria. Precipitation of phage particles in certain studies was achieved using polyethylene glycol, and the phage’s activity in the supernatant was confirmed by spot assay/DLA on different agars specific for the host bacteria. Purified phages were stored in aliquots of phage buffer and glycerol or Luria-Burtani (LB) broth at 4 °C for temporary storage or at −80 °C for extended storage. Protocol variations between reviewed studies include centrifugation speed ranging from 3000× *g* to 8000× *g*, precipitant used, the addition of cesium chloride (CsCI), which blocks bacterial lysin activity, and the phage storage solution used.

### 3.4. Effectiveness of Phage Therapy as a Treatment Option

Among the studies analyzed, phage therapy’s effectiveness was investigated predominantly in three clinical manifestations, pneumonia, biofilm, and bloodstream infections such as bacteremia and sepsis. In the 29 reviewed studies, 28 unique phage and phage cocktails were used to target pneumonia (seven studies), biofilms (nine studies), bacteremia (six studies), and sepsis (one study) and one study that analysis the role of phages in both biofilm and bacteremia model. In the sections ahead, these three prominent clinical manifestations will be discussed further, and the effect of phage therapy in these will be addressed.

#### 3.4.1. Pneumonia

Among the seven studies that evaluated phage therapy against pneumonia, three studies targeted *P. aeruginosa* and two studies each evaluated *S. aureus* and *K. pneumoniae* respectively [18,19,24,27,30,31,39]. All seven studies showed the effectiveness of phage therapy against *P. aeruginosa, S. aureus,* and *K. pneumoniae* with a remarkable reduction in bacterial cell population and a significant improvement of survival to varying degrees in a dose-dependent matter. In one of the studies evaluating multiple phages, via intranasal approach to administer phage therapy against pneumonia, two of the nine tested phages had an insignificant improvement of mice’s health and the bacteria were able to develop resistance, which occurred spontaneously 24 h post *P. aeruginosa* infections [27]. Out of the seven studies, only one study assessed the effect of combination therapy of phage with antibiotics (teicoplanin) administered intravenously to target *S. aureus.* The combination therapy did not improve the treatment outcome as no synergistic or antagonistic effects were observed [39]. Four studies reported phage therapy’s effect on cytokines level (TNF-a and IL-6), and a significant reduction was observed in the phage treated model compared to the control [19,24,30,39]. One study reported lowering of Lower IFN-γ, TNF-a, IL-1α, and IL-8 by phage therapy [31].

#### 3.4.2. Biofilm

Nine studies assessed phage as a treatment option against biofilm formation. Among these studies, one article targeted biofilm produced by *P. aeruginosa*, two by *S. aureus*, three by *E. faecalis*, and one by *K. pneumoniae*. One study tested the effect of phages on dual infections caused by *S aureus* and *P. aeruginosa* and one tested their effect on both organisms individually [34,43]. In all the reviewed studies, bacteriophage was active against both planktonic cells and biofilm, showing a bactericidal effect and detachment activities against tested pathogens. Out of the nine studies, two evaluated the effect of combination therapy of phage with antibiotics against *S. aureus* using vancomycin/rifampicin, and teicoplanin, respectively [35,43]. The first *S. aureus* study illustrated a synergistic effect between vancomycin/rifampicin, and bacteriophage when phage was administered directly to tissue culture plates at a low concentration of phage (10^5^ PFU/mL) and vancomycin (6 mg/L)/rifampicin (MIC of 0.016 mg/L) [35]. At higher concentrations, no synergy was seen. In the second study against *S. aureus*, teicoplanin administration combined with phage resulted in no visible biofilm in in vivo experiments [43].

Additionally, although the administration of teicoplanin in combination with phage degraded biofilm produced by *S. aureus*, the method of phage (intravenous) and antibiotics (Intraperitoneal) administration was different, which lacked justification in the paper [43]. One of these studies also tested the effect of combination therapy of phage with imipenem, cilastatin, and amikacin against *P. aeruginosa* in an in vivo biofilm model [43]. In this study, although both bacteriophage and bacteriophage plus antibiotic combination reduced bacterial load in the animal, they did impact biofilm thickness. Another study compared the activity of phages vs. antibiotic (tetracycline) against *S. aureus* and *P. aeruginosa* dual infection biofilm where tetracycline showed superior anti-biofilm activity than the tested phages [34].

Additionally, an *E. faecalis* study illustrated that a phage isolated from wastewater was able to target and degrade the biofilm at first, but then the bacteria were able to develop resistance against the selected phage. This was indicated by the resistance mutation in the enterococcal polysaccharide antigen gene (*epa*), which is necessary for the infectivity of phage [22].

Although all reviewed studies showed significant degradation and bactericidal effects on biofilm, the mechanism by which phage can disperse biofilm is yet to be explained. Possible mechanisms include the expression and secretion of depolymerizing enzymes by phages that degrade extracellular polymeric substances (EPS) or because of potentially high capacity of phages that allow it to infect metabolically inactive persister cells found within biofilms. These are topics that need further investigation.

#### 3.4.3. Bloodstream Infections: Bacteremia and Sepsis

From the 29 reviewed studies, five articles assessed the impact of phage therapy on bacteremia in *P. aeruginosa* (16.7%) [17], *E. faecalis* (16.7%) [20], *K. pneumoniae* (33.3%) [28,29], and *S. aureus* (16.7%) [41], and one tested the efficacy of phage against sepsis model specifically against *E. faecalis* [44]. One particular study tested both host specific phage against *K. pneumoniae* and *P. aeruginosa* infections individually and the combined phage cocktail against both single and polymicrobial infections caused by these two bacteria [32]. All studies showed high efficacy of phage treatment by rescuing the model used from death caused by bacteremia. Among the six studies, two evaluated the effect of delayed treatment on the bacteremia model used. The results illustrated that delayed treatment could reduce the protection rate as seen by differences in survival rate between page therapy at 10 min (100% survival) vs. 1 h (12.5% survival) after infection in one of the studies [29] and between survival rate at 4 h (90% diabetic mice and 100% non-diabetic mice) and survival rate at 8 h (90% diabetic mice)/20 h (0% non-diabetic mice) in the other study [44]. In the other study which tested delayed treatment effect, a negative impact on the health score of the animals was seen in response to treatment delays (18 h after bacterial challenge), but this did not impact the survival rate with the eventual recovery of health back to normal within four days [32].

In comparison to antibiotics, one study that tested oxacillin efficiency vs. phage on *S. aureus* infection reported significantly less viable bacteria when phage was administered vs. control in contrast to antibiotic (oxacillin) treatment [41]. Of interest, no studies noted adverse events with phage administration, and only one study data reported the emergence of bacterial resistance against the tested phage [29]. Most of these studies did not evaluate the combination of phage therapy with antibiotics or critically evaluate resistance development and associated mechanisms. Only one study reported phage therapy’s effect on cytokines level and identified statistically significant lower TNF-α, MCP-1, IL-10, and IL-6 upon phage therapy [29]. Only one study out of the 29 reviewed studies evaluated phage therapy’s effect on *E. faecalis* infections’ sepsis model. In this study, both the phage as well as the phage lysin and both phages and the endolysin reduced bacterial load and protected mice from lethal challenges (survival rate of 60–80%).

### 3.5. Phage Safety and Efficacy

Among the 29 reviewed studies, many investigated the phage’s safety and efficacy in successfully reducing bacterial growth and enhancing clinical outcomes without significant side effects. Despite the studies using different dosages, phage administration routes, and different infection models, no adverse effects were reported.

## 4. Discussion

In this systematic review, available data were critically selected and evaluated to establish the current use of phages as therapeutic options on selected WHO priority list pathogens. Most of the reviewed studies showed that various phage cocktails or phages had a high efficacy level against some of the selected pathogens and were safe, as indicated by the absence of side effects. It is critical to note that many of the models used were infected with bacterial strains showing multi-drug resistance to the available antibiotics commonly used for the treatment. In all of these cases, bacteriophage effectively reduced the bacterial concentration, improved outcomes, and protected from lethal infections or degraded biofilms. Only in two of the studies, partial failures were met when 2 of the tested phages failed to protect the animals from infection, and one of the phages did not display a significant anti-biofilm activity, respectively [27,43].

### 4.1. Appropriate Phage Selection for Therapy

From all the studies reviewed, it is well-established that appropriate phage selection and the host range of phage used are among several elements that must be considered for phage’s therapeutic applications. For better therapeutic efficacy, selected phages must be safe, polyvalent, strictly lytic, able to replicate in the host, stable, and preferably show synergistic therapeutic activities combined with antibiotics or other phages as a phage cocktail to limit bacterial resistance development. Almost all of the discussed studies in this review reported no side effects related to phage therapy, which could be justified by the fact that phages mostly consist of protein and nucleic acid (DNA or RNA) that do not initiate toxic or allergic reactions in the model used and target only specific bacteria. Additionally, the purification method and dilution of administered phages would further enhance their safety, eliminating bacterial toxins and lysates, thereby avoiding potential side effects and immune response.

On the other hand, some phage genomes might carry drug resistance or virulent genes, which would make them unstable for successful therapy. To eliminate the possibility that such genetic elements exist in the phage genome, scientists could use naturally occurring strictly lytic phages. These phage’s genomes do not integrate with bacterial genomes and do not carry or propagate virulent and resistance genes between bacteria [46]. The small genome size combined with reducing sequencing costs also makes phages extremely amenable to sequencing, which can be further used to identify the presence/absence of resistance elements in these phages and serve as a guide for appropriate phage selection. Genetically engineered phages offer another avenue of customizing phage genome to suit infection models or treatment regimens, and these phages were efficient as seen in one of the reviewed studies in Table 1 where a genetically engineered phage was able to reduce bacterial titer by 99% in case of vancomycin-resistant infection in an in vitro model [42]. In the case of phage resistance, several polyvalent phage cocktails may be prepared and used in alternation.

### 4.2. Challenges of Phage Therapy

Bacteriophages therapy implementation as a therapeutic approach faces three main challenges: (i) manufacturing; (ii) availability of limited published data, which makes the application challenging, and lastly, (iii) difficulties in regulatory processes. A systemic approach regarding phage’s natural occurrence, efficacy, and safety should be obtained to promote their development and encourage their acceptance as a therapeutic option against bacterial infections. Moreover, phage manufacturing (either as a single or phage cocktail), including isolation, preparation, and propagation, should adhere to Good Manufacturing Practice (GMP) guidelines, as an effective phage therapy relies majorly on maintaining phage stability and reducing immune reaction from the manufacturing process to the administration time. Although the Food and Drug Administration mandates that all phages be sterilized and highly purified before usage to minimize potential side effects, more effort is required to standardize the purification method based on the phage host range, thus minimizing the risk of observed variability between experimental and clinical trials results. Finally, the establishment of a simple regulatory process is required for the clinical use of phage. Bacteriophage therapy is considered as a personalized approach, targeting specific pathogens for each patient. As such, phages are then a customized therapeutic product that suits each case independently instead of a fixed general medicinal product. One possible regulatory approach for phage therapy may include the multi-strain dossiers used previously, with some vaccines covering several different strains [47]. This approach follows a fast and frequent modification policy that might reduce the risk of resistance development. Agreement and communication between the regulatory authorities, researchers, drug developers, and safety agencies are required for better-personalized phage therapy outcomes. Other challenges include the rapid clearance of phages from the circulatory system by many mechanisms, such as bacteriophage exclusion which enables selective methylation of host genome thus preventing the propagation of phage and the rapid lyse by phage of many targeted bacteria, which might secrete endotoxins that could evoke a massive immune response.

### 4.3. Recommendations for Successful Phage Therapy

A successful bacteriophage therapy depends mostly on the type of infection (superficial, chronic, or systemic infection), causative agent, phage administration’s timing (before or after the infection), and the animal/model used/biofilm condition. To be clinically relevant, appropriate animal models need to be used, which need to mimic real-life patient’s clinical settings and phage properties closely. Several factors must be considered when using bacteriophages as a treatment option. First, the clinical conditions of the treated model/patient may dictate the duration and the effectivity of the treatment. For instance, an extended treatment period may be required based on the type and site of infection or when the model/patient has multiple comorbidities. In the literature pertaining to phage therapy, depending on the severity of infection and phage administration time, the models used in various studies required days to months of phage therapy to eradicate or alleviate the infection. In the case of *P. aeruginosa*, bacterial resistance developed within 2 h after phage therapy [25]. These two points illustrate that timing is critical as resistance is a real possibility and can develop spontaneously after administration.

Second, the dosage and administration routes of phages. Selecting the appropriate course of administration and calculating the correct phage dosage requires an accurate estimation of severity and type of infection and the model/patient clinical conditions. For example, when treating a septic case caused by a wound infection, spraying the phage preparation on a wound’s surface might not be as effective and efficient as gel preparation due to adherence problems. Additionally, although shown to be effective in two studies reviewed here [26,29], oral administration of phages might be challenging as some phages may become inactivated in the proteolytic and acidic stomach environment. Intravenous phage administration was seen to be effective in one of the reviewed studies [39]. However, it can lead to cytokine storm by rapidly eradicating bacterial pathogens and triggering the immune response to severe outcomes by developing phage specific T-cells and the release of cytokines. However, no side effects were reported in the reviewed study here. In 34.5% of the studies included in this review, intraperitoneal phages administration was commonly used, but limited data is available on the applicability, practicality, and safety of this approach in humans [17,20,26,29,30,32,37,41,44]. Intranasal administration showed significant improvement of pneumonia in all five studies [18,19,24,27,31]. All the reviewed studies used sensitivity assay, bacteriophage adsorption assay, platting efficacy, and minimal lethal dose concentration studies to determine a phage dosage-concentration required for treatment.

To successfully implement phage therapy, collaborative efforts are required to standardize the model used, select the appropriate administration route depending on model and type and site of infection, and determine the frequency and dosage of administered phage. It is critical to develop a novel strategy to estimate the effective administration time, thus enhancing treatment outcomes and reducing resistance risk. Ultimately, the success of phage therapy could be improved by evaluating phage pharmacology (pharmacokinetics and pharmacodynamics), targeting phage resistance mechanisms, and derived proteins.

### 4.4. Combination Therapy: Is It the Solution?

Combination therapy could be used in clinical settings to successfully treat and prevent or reduce bacterial resistance development via a synergistic effect. Treatment synergy occurs when the combined treatment effect using two or more agents is greater than the sum of individual effects of those agents resulting in a higher treatment success rate [48]. The phage antibiotic synergy (PAS) approach was used to illustrate that sublethal concentrations of antibiotics could enhance bacterial production of lytic phages. This works likely by the low dosage of antibiotics inhibiting bacterial cell division and increasing biomass leading to shorter latent period and increased burst size of phages allowing the phages to destroy the remaining bacterial cells faster [49].

In this systematic review, five studies were included in which combined therapy of antibiotics and phage therapy were tested [21,35,37,39,43]. All five studies dealt with *S. aureus* infections, with one of the studies, also including *P. aeruginosa*. The *S. aureus* studies included systemic and localized burn wound infection model [21], in vitro biofilm [35], peri-prosthetic joint infection model [37], ventilator-associated pneumonia [39], and implant-related osteomyelitis model [43], and phages and antibiotics were simply added in cased in vitro model [35]. In vivo studies saw phages being administered subcutaneously/through the skin in two studies [21,43], intraperitonially and intravenously in one study each, respectively [37,39]. Antibiotics were administered in the animal studies either orally [21], intraperitonially [37,43], or intravenously [39]. The results of combination therapy were mostly synergistic, reducing bacterial concentration significantly. The treatment outcome of combination therapy for *S. aureus* infections was synergistic in four of the five studies and included a significant improvement in survival, protection from lethal infection, degradation of biofilm, or reduction in bacterial load concentration. On the other hand, one study that administered phage intravenously illustrated that combination therapy with teicoplanin did not further improve the outcome of pneumonic mice infected with *S. aureus* [39]. This shows that administration methods or dosage, or even choice of antibiotic can all affect the practicality of the approach. In the case of *P. aeruginosa* infections model, combination therapy successfully reduced the bacterial burden in implant-related osteomyelitis model but did not impact biofilm thickness [43].

Though using combination therapy would have potential benefits by limiting the dosage of antibiotics used, reducing the risk of antibiotic resistance development, several considerations still need to be addressed. First, successful combination therapy may require several exposures to phages over a more extended treatment period to avoid bacterial resistance and decrease the bacterial load. Additionally, considering PAS therapy’s potential, it is critical to question the degree of antibiotic interference with the phage’s bactericidal and retention functionality. Together, these findings highlight the idea that bacteriophage therapy need not aim to replace antibiotics; instead, they can work exceedingly well in combination with antibiotics against complicated infections. Limited studies are available in this field, and more studies are required to fully comprehend the full treatment dynamic for PAS combination therapy to be successfully used in clinical use.

### 4.5. Immunological Aspects of Phage Therapy

Immunological responses of the human body due to phage exposure are an essential aspect to be considered in phage therapy. Bacteriophages are present predominantly in the human body as part of the human healthy virobiota/microbiota, implying that the host immune response might not recognize phages as a threat [50]. Clear evidence in the literature is not yet available regarding the immune response toward phages. It is still not clear whether phages can evoke and interact with the host immune system. One of the reviewed studies show that in an in vivo pneumonia model of infection, administration of phages in case of infection led to a concentration of phages in the lungs, and in the absence of infection, phages were not detected in the lungs indicating that the immune system is clearing them off [39]. Another study supporting this used a mutant phage and illustrated that phages were removed from the circulation by the innate immune system [51]. Furthermore, a study concluded that phage administration mode is critical and could reduce anti-phage response during phage therapy [52]. Screening additional literature, it was also identified that some studies are illustrating the potential possibility of phages being immunomodulators, especially during the absence of bacterial infections, supported by the reduced level of proinflammatory cytokines and proteins such as C-reactive proteins, IL-6, and IL-1 and reduced cellular infiltration after phage administration [50].

In the 29 reviewed articles, eight studies examined phage therapy’s effect on some of the proinflammatory cytokines and or CRP [19,24,29,30,31,37,43]. All except 2 showed a significant reduction in their levels a few hours after phage administration [19,24,29,31,43]. 4 of these studies looked at the effect of phage alone without infection, and no significant difference was seen on cytokines upon phage administration as opposed to control mice [24,29,30,39]. As phages are considered part of the healthy flora, it is not surprising nor inconvenient that phages act as probiotics and like immunomodulators. This also raises the question of whether phage therapy’s success depends on its antibacterial effect and its anti-inflammatory response, making it critical to investigate the effect of phage alone without infection on the host immune system. Additionally, limited studies are available to demonstrate how anti-phage cellular mechanisms occur or how phages are presented to T-cells.

### 4.6. Limitations

In this systematic review, a few limitations of the reviewed studies could be identified. Firstly, a few reported studies lacked a detailed explanation about the inflammatory response, the safety of the phages used, and the side effects developed; however, they were included in this review due to their significant impact and uniqueness in other aspects. Second, reported studies did not have a uniform description of outcomes; although *p*-values were used in all papers to estimate the significance of the treatment, another description needs to be established to assess different sites and types of infections. Additionally, although multiple clinical trials have been published, we excluded all of them from this study to understand the potential reasons behind their low success rates, as experimental studies represent the foundations for clinical trials.

In light of the rising problem of antibiotic resistance worldwide, phage therapy seems to be a safe and effective strategy to combat the impact of bacterial resistance. However, there is a shortage in the number of studies that comprehensively assess phage therapy’s safety and efficacy. Moreover, available studies lack a transparent and standardized methodology for isolation and purification of phage, resulting in significant inoculum and outcome variations between studies despite using the same model, phage, type of infection, and assay. Finally, phage therapy’s effect on an immune response needs more elaboration to ensure phage therapy’s effectiveness.

### 4.7. Future Directions

Since the advances in molecular microbiology, scientists have been better able to understand biological properties of bacteriophage’s and appreciate the complexities of their interactions with both the bacteria and the host. However, despite our knowledge of phage’s antibacterial activities and their manipulations in vitro, limited information of their activities in vivo is available, specifically through clinical trials. Although many phage therapy study outcomes were identified as safe, not all have shown effectiveness against bacterial infections. To better understand phages, controlled, methodical experimental designs are essential to evaluate phage usage as therapeutic agents adequately. More attention should be given to isolation, formulation, purification, and delivery of phages to minimize any side effects that might result from an immune response to contaminants or carriers. Possible ways to enhance phage’s delivery system could include using liposome (phospholipids) as a delivery vehicle [53].

**Figure 2:**
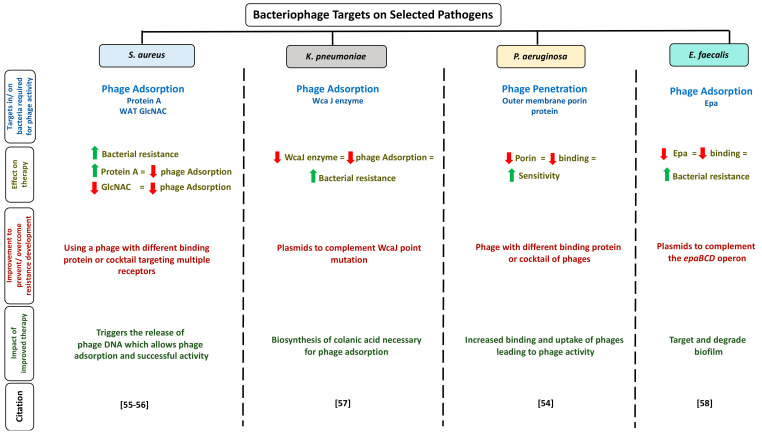
Schematic illustration of selected bacteriophage targets available in the literature. The figure is divided into four columns and five rows. The column headings represent the pathogens, while the rows heading represent targets in/on bacteria required for phage activity, effect of these targets of phage therapy, available methods to improve target to prevent/overcome phage resistance development, the outcome observed if the improved method is used, and citation. Abbreviations: WAT GlcNac: wall teichoic acid N-acetylglucosamine; WcaJ enzyme: undecaprenyl-phosphate glycosyltransferase; Epa: enterococcal polysaccharide antigen. See Refs. [55,56,57,58].

Furthermore, scientists could use phage therapy to manipulate bacterial behavior against antibiotic resistance. Phages target specific surface components/processes within bacteria of the cell wall during their activity, and some of these are also utilized by the bacteria for mediating antibiotic resistance, such as outer membrane porins and efflux pumps. For instance, it has been shown in previous studies that therapy with phages that bind to outer membrane porins inhibited the antimicrobial resistance activity mediated by these porins, and this, in turn, increased the sensitivity of these bacteria to various antibiotics [10,54]. Phage therapy can also exert selective pressure on the target, such as the bacteria undergo mutations in the target for developing phage resistance at the cost of losing the antimicrobial resistance activity. For example, usage of a specific phage that binds to outer membrane porin M that is also involved in multi-drug efflux. In response, bacteria may mutate or change porin M structure or function to reduce resistance and enhance antibiotic sensitivity. In other words, using phages to target specific parts in bacteria to reduce antibiotic resistance indirectly instead of directly targeting the whole microorganism. Various targets in bacteria were identified as required for phage activity. Figure 2 summarizes some of the identified targets of phage on selected pathogens. Moreover, identifying available phage targets in bacterial hosts could also provide better insights into phage resistance development by bacteria and ways to overcome it Figure 2. For example, with *K. pneumoniae*, WcaJ, an initial enzyme catalyzing the biosynthesis of colanic acid is necessary for phage adsorption to complete its lytic life cycle. A frame deletion of *wcaJ* provided phage resistance in the wild type strain. Using plasmids amplified using polymerase chain reaction and cloning to complement this mutation could demonstrate phage susceptibility which could increases current understanding of anti-phage defense mechanisms in these pathogens. Moreover, with *P. aeruginosa*, using phages with serine protease binding proteins could digest porin protein on the bacterial outer membrane, therefore, reducing bacterial binding and enhancing their sensitivity to therapy.

Multiple researchers believe that phage therapy falls under personalized medicine. This allows this therapeutic approach to be more specific towards host strains. Since multiple strains of pathogenic bacteria are present, and phages are known to be very specific for strains, a profiling system of well-characterized phage assigned to their hosts would be needed to enhance personalized treatment. Lastly, collaborations between scientists, researchers, academics, students, and governments are needed to establish a safe and effective foundation for phage therapy. 

### 4.8. Clinical Trails Current Progress

The field of phage therapy is rapidly evolving and has resulted in cases with successful safety outcomes and failures. A randomized phase two clinical trials against *P. aeruginosa* illustrated that phage administration at low concentration (1 × 10^2^ PFU/mL/day) reduced bacterial concentration slower pace compared to standard treatment [59]. Previously, a single-arm non-comparative trial against *S. aureus* reported the effective and safe effects of phage’s intravenous administration in thirteen patients [60].

However, one of the challenges with phage therapy is the lack of sufficient clinical trials [61]. A systemic review published in 2019 evaluated available clinical trials against multidrug-resistant pathogens from 1985 to 2018 [62]. The study highlighted the importance of policies and regulations and standardization at a national level, which might help introduce phages to clinical practices. Table 2 lists the recent phage therapy clinical trials registered in https://ClinicalTrials.gov.

### 4.9. Microbiome and Bacteriophages: Their Correlations?

The gut microbiome is known to have a significant impact on health outcomes. Bacteriophages dominate the human gut microbiome’s viral component, which plays an essential role in driving bacterial diversity and shaping microbial composition [69]. Despite the presence of bacteriophages in the gut, the administration of therapeutic phage could act as an external agent that could promote species exclusion by predation with indirect consequences on the gut metabolome [70,71]. Limited reports are available on the effect of phage predation on the gut metabolome. Some reports claim that phage treatment induces minimal composition changes, and others reported significant compositional changes [71,72]. Variations between reports could be due to model differences as well as phage host interactions. Many more studies are needed before we can use phages to modulate the gut microbiome, especially ones that prove that the approved therapeutic phage will not have the ability to integrate its genome into the microbiome niche genome or persist long enough to affect microbiome through strong selective pressure indirectly.

## 5. Final Thoughts

The rapid and ever-growing increase in numbers of antibiotic resistance pathogens calls for immediate alternative treatments. Phage therapy represents a well-suited option to be included in the multidimensional strategies to combat it due to its diversity and adaptability. This review highlighted the significance of phage therapy and addressed the work needed in the clinical, experimental, manufacturing, and regulatory field to emphasize phage’s value as an antibacterial agent. Although the field of phage therapy is rapidly advancing, gaps in the knowledge need to be addressed and filled. To effectively implement this approach, phage therapy applications should not aim to replace antibiotics, but instead complement their effects against infections. By accepting this, efforts will be put into safety and efficacy evaluation, protocol standardization, and host range identification. Finally, although phage therapy has multiple challenges, undertaking it will improve treatment outcomes.

## Figures and Tables

**Table 1:** Summary of the main features retrieved from 29 reviewed articles.

Serial Number	Target Bacteria	Model Type/Age/ Gender	Phage/Cocktail Identification	Site/Type of Infection	Method of Inoculation	Follow Up Period	Therapy Dose	Method of Testing	Immune Markers	Outcome	Combination Therapy	Emergence of Resistance	Ref.
1	*S. aureus* (Methicillin resistant)	Plates	Phage K and DRA88 (*Myoviridae* family) from sewage alone and in a cocktail	Biofilm	Addition to plate	1–48 h	10^6^–10^7^ PFU/mL	Electron microscopy Genomic analysis Biofilm assays	Not assessed	Phage cocktail was able to disrupt biofilm	Not assessed	Not assessed	[16]
2	*P. aeruginosa*	Specific-pathogen-free (SPF) female mice, 6–8 weeks old	RLP phage from water body (*Podoviridae* family)	Bacteraemia	Intraperitoneal	21 days	1 × 10^9^ PFU	Electron microscopy Genomic analysis Survival tests	Not assessed	Mice were rescued from bacteraemia (92% survival rate)	Not assessed	Not assessed	[17]
3	*K. pneumoniae*	BALB/c mice 6–8 weeks old	VTCCBPA43 from water body (*Siphoviridae* family)	Pneumonia	Intranasal	10 days	2 × 10^9^ PFU	Nano-scale liquid chromatography–tandem mass spectrometry Electron microscopy Animal studies and histopathology	Not assessed	Significant reduction of bacterial load in lungs	Not assessed	Not assessed	[18]
4	*K. pneumoniae*	Webster female mice 7 weeks old	Phage 1513 (*Siphoviridae* family)	Pneumonia	Intranasal	72 h	2 × 10^9^ PFU/mouse	Electron microscopy Histopathology ELISA	Lower TNF-α Lower IL-6	Phage improved lung lesions and 80% survival rate	Not assessed	Not assessed	[19]
5	Vancomycin-resistant *E. faecalis*	BALB/c female mice 6–8 weeks old	EF-P29 phage from sewage (*Siphoviridae* family)	Bacteraemia	Intraperitoneal	2 days	4 × 10^5^ PFU	Electron microscopy Genomic analysis Survival tests	Not assessed	Protection of all mice from bacteraemia (100% survival)	Not assessed	Not assessed	[20]
6	*S. aureus* (Methicillin resistant)	BALB/c female mice 4–6 weeks old	Endolysin from MR-10 phage	Systemic burn wound infection	Subcutaneously near the site of burn wound	7 days	50 ug/mL	Histopathology Survival tests	Not assessed	Combination therapy of endolysin plus antibiotic found to be effective against systemic infection (100% survival)	Minocycline	Not assessed	[21]
7	*E. faecalis* (multiple strains)	Plates	EF1TV phage from sewage (*Herelleviridae* family)	Biofilm	Addition to plate	5 days	10^9^PFU	Electron microscopy Confocal microscopy Biofilm degradation	Not assessed	Exhibits anti-biofilm activity (9–68% reduction in different *E. faecalis* strains)	Not assessed	Yes	[22]
8	*P. aeruginosa*	Plates	BrSPI phage from sewage (*Caudovirales* family)	Culture	Addition to culture	24 h	10^6^PFU/mL	Electron microscopy Genomic analysis Biological in vitro assays	Not assessed	Effective for controlling bacterial growth until 12 h post-infection	Not assessed	Yes	[23]
9	*P. aeruginosa*	Balb/c male mice 8 week old	PAK-P1 phage from sewage (*Myoviridae* family)	Pneumonia	Intranasal	12 days	10^8^ PFU	Electron microscopy Inflammation analysis Genomic analysis Survival tests Luminescence assay	Lower TNF-α Lower IL-6	Protection from lethal pneumonia (100% survival)	Not assessed	Not assessed	[24]
10	*P. aeruginosa*	Hydrogel-coated catheters.	Cocktail consisting of phages from sewage combined with phage M4	Biofilm	Exposing the catheter lumen to phage lysate	2 days	7.0 × 10^9^ PFU/mL	Biofilm degradation assay Electron microscopy	Not assessed	Phage cocktail pre-treatment significantly controls biofilm formation	Not assessed	Yes	[25]
11	*P. aeruginosa* (multidrug resistant)	BALB/c male mice 7 weeks old	Lytic phage PS5 from clinical specimen (*Myoviridea* family)	Acute and chronic wound infection model	Intraperitoneal and orally	7 days	9 × 10^8^ PFU, administered 30 min after bacterial challenge and 24 h after the first injection, and then given a daily dose of phage orally (3 × 10^8^)	Electron microscopy PCR Survival tests	Not assessed	Resolution of infection in acute wound model and complete recovery of the infected rodents in chronic wound infection model.	Not assessed	Not assessed	[26]
12	*P. aeruginosa*	BALB/c male mice 8 weeks old	Multiple bacteriophages from sewage tested individually (*Myoviridea* and *Podoviridae* family)	Pneumonia	Intranasal	13 days	10^6^ PFU	Survival tests Genomic analysis Luminescence assay	Not assessed	37–100% survival rate with seven of the nine tested phages at the indicated dosage in *P. aeruginosa* PAK-lumi strain. Two strains did not show sufficient activity.	Not assessed	Not assessed	[27]
13	*K. pneumoniae* (Carbapenem resistant)	Plates and BALB/c female mice 6–7 weeks old	vB_KpnS_Kp13 from sewage (*Siphoviridae* family)	Biofilm and in vivo infection model	For biofilm degradation assay, phages were added to the plates Intraperitoneal injection for mice	2 days (biofilm) 10 days (in vivo)	2 × 10^8^ PFU/mL 1.75 × 10^8^ PFU/mice	Electron microscopy Confocal microscopy Genomic analysis Biofilm degradation Survival assay	Not assessed	Phage was able to degrade 70% of biofilm Phage administration within 10 min of bacterial challenge resulted in 100% survival	Not assessed	Not assessed	[28]
14	*K. pneumoniae*	C57BL/6 male mice 6–7 weeks old	NK5 from sewage (*Podoviridae* family)	Liver abscess model and bacteraemia	Intraperitoneal (IP) Intragastric (IG)	9 days	2 × 10^7^ PFU/mL (IP) 2 × 10^6^ PFU/mL (IG)	Survival assay Inhibition assays	Lower TNF-α, MCP-1, IL-10 and IL-6	Phage treatment 30 min after bacterial challenge protected mice from death (100% survival), no detectable bacteria in blood	Not assessed	Yes	[29]
15	*P. aeruginosa* (Extensively drug-resistant)	Female C57BL/6 mice 7–8 weeks old	B-R656 and B-R1836 (*Siphoviridae* family) from sewage tested individually	Pneumonia	Intraperitoneal	12 days	10^9^ PFU/mL	Electron microscopy Histopathology Cytokines assay Survival assay	Lower TNF-α IL-6	Both phages decreased bacterial load in lungs, increased survival rate (66% and 83% respectively)	Not assessed	Not assessed	[30]
16	*S. aureus*	Female rabbit	VB-SavM-JYlOI phage from sewage (*Myoviridae* family)	Rabbit necrotizing pneumonia model	Intranasal	8 days	3 × 10^9^ PFU	Electron microscopy Histopathology Cytokines assay Genomic analysis Survival assay	Lower IFN-γ, TNF-α, IL-1α and IL-8	Single dose of phage protected rabbit from pneumonia (survival rate 90% at 48 h)	Not assessed	Not assessed	[31]
17	*K. pneumoniae and P. aeruginosa*	Albino mice 1 month old	monovalent and polyvalent phage preparation	Bacteraemia	Intraperitoneal	20 days	*K. pneumoniae*: 10^8^ PFU *P. aeruginosa*:10^9^ PFU	Dose ranging assay Delayed treatment Survival assays	Not assessed	Phage cocktails were effective in rescuing mice from death in 100% of mice in case of both monomicrobial and polymicrobial bacteremic mice	Not assessed	Not assessed	[32]
18	*E. faecalis*	Plates	EFDG1 from sewage (*Myoviridae* family)	Biofilm and ex vivo two chamber bacterial leakage model of human teeth	Addition to plate (biofilm) Teeth were irrigated with phages	7 days (biofilms) 48 h (tooth model)	1 × 10^7^ PFU (biofilm) 1 × 10^8^ PFU/mL (tooth irrigation)	Electron microscopy Confocal microscopy Biofilm degradation Viability assays	Not assessed	* Effective lytic activities against biofilm: 5-fold reduction in biomass and 5 log reduction in viable counts * protects root canals from infection: 7-log reduction in viable counts	Not assessed	Not assessed	[33]
19	S. aureus and P. aeruginosa mixed infection	Plates	Phage cocktails AB-SA01 consisting of staphylococcal phages designated J-Sa-36, Sa-83, and Sa-87 (Myoviridae family) and AB-PA01 consisting of Pa-193 and Pa-204 from Myoviridae family and Pa-222 and Pa-223 from Podoviridae family.	Biofilm	Addition to plate	24 h	Not specified	Confocal microscopy Biofilm assay	Not assessed	* Individual phage cocktails and combination of the two phage cocktails produced significant biofilm biomass reduction in mixed species * Tetracycline was more effective than the phage therapy in reducing biofilm biomass.	Comparison of phage activity against Tetracycline	Not assessed	[34]
20	*S. aureus* (methicillin susceptible)	Plates	Cocktail of three phages: PP1493, PP1815, and PP1957 from sewage	Biofilm and in vitro model of osteoblast infection	Addition to plate	24 h	10^8^ PFU	Biofilm assay	Not assessed	Cocktail active against mature biofilm with 3.6 log reduction in viable bacteria count -Synergistic effect between antibiotics and bacteriophages at lowed doses was observed -bacteriophages had no intracellular activity	Vancomycin/Rifampicin	Not assessed	[35]
21	*P. aeruginosa*	Liquid culture	PaPI phage	Liquid culture	Addition to culture	Not specified	10^10^ PFU/mL	Biofilm assays Confocal microscopy Genomic analysis	Not assessed	Lysed cells	Not assessed	Yes	[36]
22	*S. aureus* (methicillin susceptible)	Sprague-Dawley male rats 20 weeks old	StaPhage cocktail consisting of 5 *Myoviridae* family phages: StaPh_1, StaPh_3, StaPh_4, StaPh_11 and StaPh_16	Peri-prosthetic joint infection model	Intraperitoneal	7 days	Three doses of 1.3 × 10^8^ PFU; 8.9 × 10^5^–1.9 × 10^6^ and >10^4^ PFU respectively	Bacterial load assay Cytokines assay	No significant difference in levels of TNF-𝛼, IFN-γ, MCP-1, IL-1ß, IL-12p70, IL-10, IL-6 and IL-4 between phage treatment and control groups in the joint tissue, MCP-1 was significantly lower in the phage plus vancomycin treatment group compared to controls	Phage cocktail treatment led to 5-fold reduction and combination treatment of phage with vancomycin led to 22.5-fold reduction in bacterial load in joint tissue compared to controls	Vancomycin	Yes	[37]
23	*E. faecalis* and *E. faecium*	Plates (biofilms) and 96 well plates (colonization assay)	Phage cocktail of vB_EfaS-Zip (Zip) (*Siphovirus* family) infecting *E. faecium* and vB_EfaP-Max (Max) *(Podovirus* family) infecting *E. faecalis* from sewage	Mixed infection collagen wound in vitro biofilm model and 3T3 cell colonization assay	Addition to plate	1 day	1 × 10^8^ PFU of cocktail (biofilm) 1 × 10^7^ PFU/mL of individual phages (colonization)	Electron microscopy Biofilm assays Cytotoxicity assays Mammalian cell infection assays	Not assessed	Dual species biofilms: phage cocktail led to reduction of cell concentration by 2.5 log CFU/mL 3T3 colonization assay: both phages individually in respective bacterial infections reduced viable bacterial cells by approx. 3 log CFU/mL in 6 h	Not assessed	Not assessed	[38]
24	*S. aureus* Methicillin resistant	Wistar male rats, 9–10 weeks old	Phage cocktail consisting of equal titers of phages 2003, 2002, 3A, and K	Ventilator associated pneumonia	Intravenous	4 days	2–3 × 10^9^ PFU/mL; 5 doses	Survival assay Histopathology Cytokines assay	Reduced TNF-α IL-6 Levels	Significant improvement in survival rates (58%) compared to absolute mortality in controls, with reduced bacterial load and better histopathological outcomes	Teicoplanin: combination therapy of both phage and antibiotic did not improve results than single agent therapy alone	Not assessed	[39]
25	Multi drug resistant strains of *P. aeruginosa, S. aureus* and *K. pneumoniae*	ln vitro in flasks	*K. pneumoniae* phage: KP DP1 *P. aeruginosa* phage: PA DP4 *S. aureus* phage: SA DP1, all isolated from sewage	Liquid culture	Addition to flask	1 day	10^9^ PFU/mL	Bacterial reduction assay in liquid medium	Not assessed	Phages applied on respective bacteria reduced bacterial load but bacteria could regrow after 14–16 h of phage therapy	Not assessed	Yes	[40]
26	*S. aureus* (methicillin sensitive)	BALB/c female mice six weeks old diabetic and non-diabetic mice	GRCS phage from raw sewage (*Myoviridae* family)	Bacteraemia	Intraperitoneal	30 days	2 × 10^9^ PFU	Electron microscopy Survival tests	Not assessed	Protection from lethal bacteraemia (survival rate 90% in case of diabetic and 100% in case of non-diabetic bacteremic animals versus 0% for controls)	Oxacillin: phage therapy showed improved outcome compared to Oxacillin	Not assessed	[41]
27	*E. faecalis* vancomycin-sensitive and resistant strains	Two-chamber device containing human dentin segments	Genetically engineered phage, ϕEf11/ϕFL1C(Δ36)PnisA	In vitro infected-dentin models	Addition to root canals	7 days	5.8 × 10^9^ PFU	Recovery and Inhibition assays	Not assessed	Titre of bacteria reduced by 18% in case of vancomycin sensitive strain and by 99% in case of vancomycin resistant infections	Not assessed	Not assessed	[42]
28	*S. aureus* (methicillin resistant); *Pseudomonas aeruginosa* (carbapenem sensitive)	Adult Sprague-Dawley albino male rats	Sb-1 phage for *S. aureus* and PAT14 phage for *Pseudomonas aeruginosa*	Biofilm (implant-related osteomyelitis model)	Injected through the skin	15 days	10^7^ PFU	Biofilm assays Bactericidal test ELISA	Lower C-reactive protein	*S. aureus*: only bacteriophage plus antibiotic therapy significantly reduced bacterial load and prevented biofilm formation *Pseudomonas aeruginosa:* both bacteriophage, and bacteriophage plus antibiotic combination reduced bacterial load but did not impact biofilm	Teicoplanin for *S. aureus* and imipenem, cilastatin and amikacin for *Pseudomonas aeruginosa*	Not assessed	[43]
29	*E. faecalis*	BALB/c female mice 6–8 weeks old	IME-EF1 phage from sewage (*Siphoviridae* family) and purified endolysin	Sepsis	Intraperitoneal	4 days	2 × 10^10^ PFU/mL/0.2 mg of expressed endolysin	Electron microscopy Survival tests Lytic binding assay	Not assessed	Both phage and the endolysin reduced bacterial load and protected mice from lethal challenges (survival rate of 60–80%)	Not assessed	Not assessed	[44]

Abbreviations: TNF-α, tumor necrosis factor alpha; IL-6, interleukin 6; MCP-1, monocyte chemoattractant protein-1; PFU, plaque-forming units; ml, milliliter; BALB, Bagg and Albino; ELISA, enzyme-linked immunosorbent assay. Phage isolation, purification, and propagation, all studies used double layer agar plate, platting efficacy, and sensitivity assay.

**Table 2:** Summary of the recent phage therapy clinical trials.

Serial Number	Trial Title	Medical Condition	Method of Intervention	Status	Country	References
1	Standard treatment Associated with Phage Therapy Versus Placebo for Diabetic Foot Ulcers Infected by *S. aureus* (PhagoPied)	Diabetic foot	Topical anti-*Staphylococcus* bacteriophage therapy	Not yet recruiting	France	[63,64]
2	Bacteriophage Therapy in Patients with Urinary Tract Infections (*E. coli* and *K. pneumoniae*)	Urinary tract infection	Biological: Bacteriophage Therapy (intravenous/intravesical route)	Not yet recruiting	United States of America	[65]
3	Phage Therapy for the Prevention and Treatment of Wound Infections in Burned Patients (*S. aureus*, *P. aeruginosa*, or *K. pneumoniae*)	Wound infection	Bacteriophage cocktail spray over burned area	Not yet recruiting	Australia	[66]
4	Antibacterial Treatment Against Diarrhea in Oral Rehydration Solution (*E. coli*)	Diarrhea	T4 phage cocktail-oral	Terminated	Bangladesh	[67]
5	Bacteriophages for Treating Urinary Tract Infections in Patients Undergoing Transurethral Resection of the Prostate	Urinary Tract Infections	PYO Phage (intravesical) installation)	Completed	Georgia	[68]

## Abbreviations

WHO	World Health Organization
*S. aureus*	*Staphylococcus aureus*
*K. pneumoniae*	*Klebsiella pneumoniae*
*P. aeruginosa*	*Pseudomonas aeruginosa*
*E. faecalis*	*Enterococcus faecalis*
PFU	Plaque forming unit
LB	Luria Burtani
CsCl	Caesium chloride
TNF-α	Tumour necrosis factor α
IL-B	Interleukin beta
IL-6	Interleukin 6
WAT GlcNac	Wall teichoic acid N-acetylglucosamine
WcaJ	Undecaprenyl-phosphate glycosyltransferase
EPS	Extracellular polymeric substances
PAS	Phage antibiotic synergy
ELISA	Enzyme-linked immunosorbent assay
DLA	Double layer agar
